# Inducible Rpt3, a Proteasome Component, Knockout in Adult Skeletal Muscle Results in Muscle Atrophy

**DOI:** 10.3389/fcell.2020.00859

**Published:** 2020-09-02

**Authors:** Yasuo Kitajima, Naoki Suzuki, Kiyoshi Yoshioka, Rumiko Izumi, Maki Tateyama, Yoshitaka Tashiro, Ryosuke Takahashi, Masashi Aoki, Yusuke Ono

**Affiliations:** ^1^Department of Muscle Development and Regeneration, Institute of Molecular Embryology and Genetics, Kumamoto University, Kumamoto, Japan; ^2^Department of Neurology, Tohoku University School of Medicine, Sendai, Japan; ^3^Department of Neurology, Shodo-kai Southern Tohoku General Hospital, Iwanuma, Japan; ^4^National Hospital Organization Iwate National Hospital, Hanamaki, Japan; ^5^Department of Aging Neurobiology, National Center for Geriatrics and Gerontology, Obu, Japan; ^6^Department of Neurology, Kyoto University Graduate School of Medicine, Kyoto University, Kyoto, Japan

**Keywords:** muscle homeostasis, Rpt3, ubiquitin proteasome system, muscle atrophy, adult skeletal muscle, sarcopenia

## Abstract

The ubiquitin–proteasome system has the capacity to degrade polyubiquitinated proteins and plays an important role in many cellular processes. However, the role of *Rpt3*, a crucial proteasomal gene, has not been investigated in adult muscles *in vivo*. Herein, we generated skeletal-muscle-specific *Rpt3* knockout mice, in which genetic inactivation of Rpt3 could be induced by doxycycline administration. The *Rpt3*-knockout mice showed a significant reduction by more than 90% in the expression of Rpt3 in adult muscles. Using this model, we found that proteasome dysfunction in adult muscles resulted in muscle wasting and a decrease in the myofiber size. Immunoblotting analysis showed that the amounts of ubiquitinated proteins were markedly higher in muscles of *Rpt3*-deficient mice than in those of the control mice. Analysis of the autophagy pathway in the *Rpt3*-deficient mice showed that the upregulation of LC3II, p62, Atg5, Atg7, and Beclin-1 in protein levels, which supposed to be compensatory proteolysis activation. Our results suggest that the proteasome inhibition in adult muscle severely deteriorates myofiber integrity and results in muscle atrophy.

## Introduction

Skeletal muscles make up approximately 40% of the total body weight, thus being the largest tissue in the body. Maintaining muscle homeostasis is essential for preserving the body’s integrity and daily function. The loss of skeletal muscle mass in humans at an older age, called sarcopenia, is a rapidly growing health issue worldwide (Vellas et al., 2013). Muscle impairment is associated with several diseases and ultimately leads to a poor quality of life. The process of proteolysis is important for the prevention of cellular dysfunction and disease progression. The regulation of skeletal muscle mass largely depends on protein synthesis and degradation processes, with the latter often associated with pathological conditions, rather than with normal cellular function. Therefore, understanding of the proteolytic system has important implications for normal maintenance of cells and tissues.

The ubiquitin–proteasome system (UPS) is the best-known cellular proteolytic system, which is responsible for degradation of the majority of misfolded or defective cellular proteins (Rock et al., 1994). Proteasomes are present in both the cytoplasm and nucleus and are enriched in nuclei of many proliferating eukaryotic cells (Enenkel, 2014; Pack et al., 2014). Also, the 26S proteasome has the capacity to degrade polyubiquitinated proteins and plays an important role in many cellular processes, such as proteostasis and transcriptional control (Durairaj and Kaiser, 2014; Gallagher et al., 2014). In skeletal muscles, excessive or defective activity of the UPS leads to detrimental effects on muscle homeostasis (Sandri et al., 2013). Therefore, protein degradation by the UPS needs to be precisely regulated to maintain muscle homeostasis.

The 26S proteasome is composed of one barrel-shaped proteolytic core complex (20S proteasome), capped at both ends with 19S regulatory complexes, which recognize ubiquitinated proteins (Baumeister et al., 1998; Tanaka, 2009). The 19S proteasome can be further divided into base and lid subcomplexes. The base has six different ATPase subunits, Rpt1–6. Rpt3, also known as PSMC4, is an essential subunit of the 26S proteasome and is required for the degradation of most proteasomal substrates. In particular, *Rpt3*-deficient mouse embryos die before implantation, owing to a defect in blastocyst development (Sakao et al., 2000), which indicates that Rpt3 plays essential roles in early embryogenesis and survival. Interestingly, *Rpt3* mutations have been described in patients with Parkinson’s disease (Wahl et al., 2008). Furthermore, a previous study has revealed that phosphorylation of Rpt3 controls cell proliferation and tumorigenesis (Guo et al., 2016). To explore the organ- and cell-specific roles of the proteasome, we generated proteasome-deficient mice by targeting *Rpt3* (Tashiro et al., 2012; Kitajima et al., 2014, 2018). In particular, we found that a conditional KO of Rpt3 in a motor neuron-specific manner resulted in locomotor dysfunction, accompanied by progressive motor neuron loss and gliosis (Tashiro et al., 2012). Moreover, we have also reported that proteasome dysfunction in *Rpt3*-deficient muscle stem cells impaired their ability to proliferate, survive, and differentiate, resulting in defective muscle regeneration (Kitajima et al., 2018).

Recently, we have also found that muscle-specific *Rpt3*-KO mice, which were generated using Cre recombinase under control of the myosin light chain 1 fast (*Mlc1f*, also known as *Myl1*) promoter (Bothe et al., 2000), exhibited proteasome insufficiency, leading to a muscle growth defect and an early death (Kitajima et al., 2014). *Mlc1f* transcripts are initially detected between E8.5 and E9.5 and are robustly expressed beginning at E10.5 (Mourkioti et al., 2008), suggesting that the *Rpt3* gene in *Mlc1f-Cre*;*Rpt3*^*f/f*^ pups can be knocked out during the embryonic stage. However, the effect of Rpt3 deficiency in the adult skeletal muscle is unknown. Here, we generated inducible muscle-specific *Rpt3*-KO mice to investigate the effect of proteasome insufficiency by the *Rpt3* gene in adult skeletal muscle.

## Materials and Methods

### Mouse Strains

The Experimental Animal Care and Use Committee of the Kumamoto University approved the animal experimentation (Ref. No. A30-098). Rpt3-floxed mice (Tashiro et al., 2012; Kitajima et al., 2014) were crossed with *ACTA1-rtTA;tetO-Cre* mice (Rao and Monks, 2009) to generate *ACTA1-rtTA;tetO-Cre; Rpt3^*f/f*^* (mKO; muscle-specific *Rpt3* knockout) mice. All mice used for these experiments were between 3 and 4 months of age.

### Doxycycline (DOX) Treatments

As described in a previous study (Rao and Monks, 2009), solutions containing 2 mg/ml DOX (Sigma) or 5% sucrose in drinking water were prepared every third day and provided to the mice to voluntarily consume for 3 weeks to induce Cre-mediated excision. While the Dox solution was provided, the mice were not permitted to drink plain water.

### Mouse Tissue Preparation

The body and wet muscle were weighed. The tibialis anterior, gastrocnemius, and soleus muscles were collected individually using standard dissection methods; cleared of excess fat, connective tissue, and tendons; and subjected to further preparation and analyses. The origins of the muscle samples (i.e., tibialis anterior, gastrocnemius, or soleus muscle) are described in each figure legend as relevant. Some portions of the muscles were frozen in isopentane and cooled with liquid nitrogen for histological and immunostaining analysis, and the other muscle portions were frozen directly in liquid nitrogen and stored at −80°C for RNA isolation or protein extraction.

### Immunostaining

Immunohistochemistry of cryosections of muscle tissue was performed as described previously (Kitajima and Ono, 2018). In brief, cryosections of the muscle tissue (10-μm thickness) were cut from the middle portion of the muscle belly. These were fixed with 4% PFA, blocked with 5% goat serum or the M.O.M kit (Vector Laboratories) for 30 min at room temperature, and incubated with primary antibodies at 4°C overnight. All immunostained samples were visualized using appropriate species-specific Alexa Fluor 488 and/or 546 fluorescence conjugated secondary antibodies (Life Technologies). Samples were then observed using an Olympus fluorescence microscope IX83 (Olympus).

### Measurement of Myofiber Diameter

For measurement of the myofiber diameter, immunostaining analysis was performed on the tibialis anterior muscle, as described in our previous study (Uchitomi et al., 2019). The primary antibodies were as follows: anti-type IIa myosin heavy chain (MyHC) (SC-71, 1:5) and anti-type IIb MyHC (BF-F3, 1:5) obtained from Deutsche Sammlung von Mikroorganismen. When stained with anti-type I MyHC antibody (BA-D5), no type I fibers were observed in the tibialis anterior muscle (data not shown). Thus, type IIa/IIb-unstained fibers were defined as type IIx fibers. Immunostained images were optimized globally and assembled into figures with Photoshop. The minimum fiber Feret’s diameter (Wang et al., 2019) was measured using ImageJ/Fiji software. Samples with significant staining artifacts were excluded from automated analyses.

### Immunoblotting

Total protein lysates were extracted from the tibialis anterior muscle for immunoblotting analysis. In brief, the tibialis anterior muscle was crushed by a homogenizer, and the crushed solution was centrifuged for 15 min at 15,000 rpm and 4°C, and the supernatant was collected. We used the BCA method to determine protein concentrations. Then, the protein fractions were extracted with a reducing sample buffer containing 5% β-mercaptoethanol and complete protease inhibitor cocktail. The protein samples (20 μg per lane) were separated on a 10–20% gradient sodium dodecyl sulfate-polyacrylamide gels and subsequently transferred to polyvinylidene difluoride membranes. The membrane was then incubated with primary antibodies. Specific signals were detected using the enhanced chemiluminescence method. Densitometry was performed using ImageJ software (National Institute of Health).

### Real-Time PCR

Total RNA was isolated using RNeasy (Qiagen). For real-time PCR, first-strand cDNA was synthesized using oligo-dT primers. The expression levels of selected genes were analyzed using the Bio-Rad CFX96 system according to the manufacturer’s instructions and quantitative PCR analysis was performed in triplicate using specific primers (Supplementary Table S1).

### Proteasome Activity

As described in our previous study (Kitajima et al., 2014), proteasome activity was assessed using Proteasome-Glo^TM^ Assay kit (Promega) following the manufacturer’s instruction. The chymotrypsin-like activity assay was conducted using skeletal muscle homogenates in a total volume of 100 ml in opaque 96-well plates. For the assays, 100 mg of protein was added to assay buffer containing 20 mM Tris-HCl (pH 7.2), 0.1 mM EDTA, 5 mM ATP, 1 mM β-mercaptoethanol, 20% glycerol and 0.04% Nonidet P40. The proteasome reagent was added separately, and 30 min later, the luminescence was recorded as relative light units on a infinite F200 pro (Tecan). Each sample was measured in triplicate.

### Antibodies

The following antibodies from Cell Signaling Technology were used: anti-GAPDH (Cat. No. 2118, 1:1000), Atg5 (Cat. No. 5840, 1:1000), Atg7 (Cat. No. 8558, 1:1000), Beclin1 (Cat. No. 3495, 1:1000), and LC3 (Cat. No. 3868, 1:200). Laminin (Cat. No. L9393, 1:100) and p62 (Cat. No. p0067, 1:1000) antibodies were obtained from Sigma. Type IIa myosin heavy chain (SC71, 1:5) and type IIb myosin heavy chain (BF-F3, 1:5) antibodies were purchased from Deutsche Sammlung von Mikroorganismen. We also used antibodies against LC3 (Novus, Cat. No. 100-2220, 1:300), ubiquitin (LifeSensors, Cat. No. VU101, 1:2000), and laminin α2 (Santa Cruz Biotechnology, Cat. No. 59854, 1:200).

### Statistical Analysis

Statistical analyses were performed with GraphPad Prism8 to determine significant differences based on a two-tailed distribution using a Student’s *t*-test. *P*-values are indicated on each figure as <0.05 (^∗^), <0.01 (^∗∗^), <0.001 (^∗∗∗^) and <0.0001 (^****^). All error bars represent means ± SEM. n.s. represents statistically non-significant.

## Results

### Generation of Muscle-Specific Rpt3-KO Mice

**FIGURE 1:**
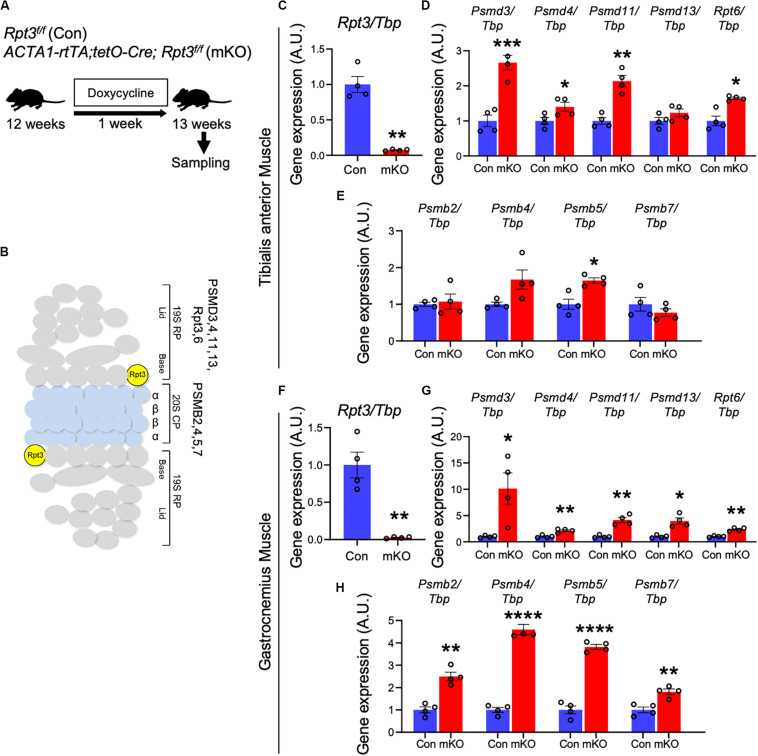
Generation of muscle-specific Rpt3-knockout mice. **(A)** Timeline of doxycycline (DOX) treatment. **(B)** The structure of the 26S proteasome. **(C)** Relative expression of *Rpt3* mRNA in the tibialis anterior muscles of Con and mKO mice after DOX treatment. The *Tbp* gene was used as an internal control. Data represent the means ± SEM (*t*-test: **p < 0.01; *n* = 4 per group). **(D)** Relative mRNA expression of 19S proteasome genes (*Psmd3, 4, 11, 13*, and *Rpt6*) in the tibialis anterior muscles of Con and mKO mice after DOX treatment. The *Tbp* gene was used as an internal control. Data represent the means ± SEM (*t*-test: *p < 0.05, **p < 0.01, ***p < 0.001; *n* = 4 per group). **(E)** Relative mRNA expression of 20S proteasome genes (*Psmb2, 4, 5*, and *7*) in the tibialis anterior muscles of Con and mKO mice after DOX treatment. The *Tbp* gene was used as an internal control. Data represent the means ± SEM (*t*-test: *p < 0.05; *n* = 4 per group). **(F)** Relative expression of *Rpt3* mRNA in the gastrocnemius muscles of Con and mKO mice after DOX treatment. The *Tbp* gene was used as an internal control. Data represent the means ± SEM (*t*-test: **p < 0.01; *n* = 4 per group). **(G)** Relative mRNA expression of 19S proteasome genes (*Psmd3, 4, 11, 13*, and *Rpt6*) in the gastrocnemius muscles of Con and mKO mice after DOX treatment. The *Tbp* gene was used as an internal control. Data represent the means ± SEM (*t*-test: *p < 0.05, **p < 0.01; *n* = 4 per group). **(H)** Relative mRNA expression of 20S proteasome genes (*Psmb2, 4, 5*, and *7*) in the gastrocnemius muscles of Con and mKO mice after DOX treatment. The *Tbp* gene was used as an internal control. Data represent the means ± SEM (*t*-test: **p < 0.01, ****p < 0.0001; *n* = 4 per group). Con indicates *Rpt3*^*f/f*^ mice, and mKO indicates muscle-specific *Rpt3*-knockout mice (*ACTA1-rtTA;tetO-Cre;Rpt3*^*f/f*^). AU, arbitrary units.

We crossed *Rpt3*-floxed (*Rpt3*^*f/f*^) mice (Tashiro et al., 2012; Kitajima et al., 2018) with a transgenic line expressing Cre recombinase under control of the skeletal muscle actin (*ACTA1-rtTA;tetO-Cre*) promoter (Rao and Monks, 2009) to generate muscle-specific *Rpt3*-KO mice. Genetic inactivation of *Rpt3* was induced in adult *ACTA1-rtTA;tetO-Cre*;*Rpt3*^*f/f*^ (mKO) mice by the administration of DOX in drinking water. DOX-treated *Rpt3*^*f/f*^ littermates were used as wild-type control (Con) mice (Figure 1A). To determine the KO efficiency of the *Rpt3* gene, we isolated the tibialis anterior muscles from Con and mKO mice after DOX treatment for 1 week (Figure 1A) and found that the *Rpt3* expression was significantly lower (*p* < 0.001), by more than 90%, in the tibialis anterior muscles from the mKO mice than in those from the Con mice (Figures 1B,C). In our previously reported *Mlc1f-Cre*;*Rpt3*^*f/f*^ KO mouse model, proteasome-related genes were upregulated (Kitajima et al., 2014). Therefore, we next investigated the expression of proteasome-related genes in the DOX-induced mKO mice. The expression of 19S proteasome-related genes, *Psmd3*, *Psmd4*, *Psmd11*, and *Rpt6*, was increased in the mKO mice compared to Con mice (Figures 1B,D), as was that of the *Psmb5* gene, which are associated with the 20S proteasome (Figures 1B,E). Furthermore, we also examined gene expression in the gastrocnemius muscle, which is mainly composed of fast-type myofibers, as well as in the tibialis anterior muscle. mKO mice showed a significant decrease in the *Rpt3* expression level and an increase in proteasome-related gene expression levels compared with those of Con mice (Figures 1F–H). By contrast, in the soleus muscle, which is mainly composed of slow-type myofibers, the *Rpt3* gene expression level was reduced by about 50% in mKO mice compared to that of Con mice, whereas the expression levels of other proteasome-related genes were not significantly altered (Supplementary Figures S1A–C). In addition, *Rpt3*-deficient mice caused a significant increase in proteasome activity in the tibialis anterior and gastrocnemius muscles, but not in the soleus muscle (Supplementary Figure S2). These results confirmed the efficiency of the muscle-specific *Rpt3* conditional KO in the tibialis anterior and gastrocnemius muscles.

### Proteasome Inhibition Induces Muscle Atrophy

**FIGURE 2:**
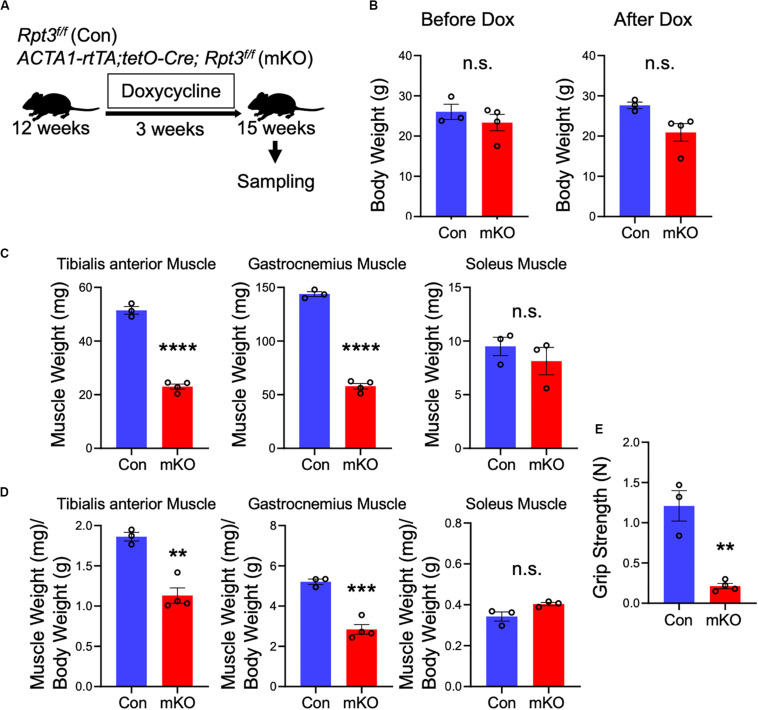
Phenotypes of muscle-specific Rpt3-knockout mice. **(A)** Timeline of doxycycline (DOX) treatment. **(B)** Comparison of body weights between Con and mKO mice before and after DOX treatment. Data represent the means ± SEM (*t*-test: n.s., not significant; *n* = 3–4 per group). **(C)** Comparison of tibialis anterior, gastrocnemius, and soleus muscle weights between Con and mKO mice after DOX treatment. Data represent the means ± SEM (*t*-test: n.s., not significant, ****p < 0.0001; *n* = 3–4 per group). **(D)** Comparison of tibialis anterior, gastrocnemius, and soleus muscle weight-to-body weight ratios between Con and mKO mice after DOX treatment. Data represent the means ± SEM (*t*-test: n.s., not significant, **p < 0.01, ***p < 0.001; *n* = 3–4 per group). **(E)** Comparison of grip strength between Con and mKO mice. Data represent the means ± SEM (*t*-test: **p < 0.01; *n* = 3–4 per group). Con indicates *Rpt3*^*f/f*^ mice, and mKO indicates muscle-specific *Rpt3*-knockout mice (*ACTA1-rtTA;tetO-Cre;Rpt3*^*f/f*^).

Next, we investigated whether *Rpt3* deficiency can cause proteasome dysfunction in adult skeletal muscles, after mouse development is completed. The mice were sacrificed, and muscle weights were determined after DOX treatment for 3 weeks (Figure 2A). There were no differences in the body weights between the Con and mKO mice before and after DOX treatment (Figure 2B). As shown in Figure 1 and Supplementary Figure S1, the *Rpt3* gene expression levels differed among the tibialis anterior, gastrocnemius, and soleus muscles; therefore, we examined the weight of each muscle. The absolute weights of the tibialis anterior and gastrocnemius muscles, but not the soleus muscles, were lower in the mKO mice (Figure 2C). Moreover, when muscle weights were evaluated per body weight, those of the tibialis anterior and gastrocnemius muscles, but not the soleus muscles, were lower in the mKO mice than in the Con mice (Figure 2D). Because proteasome-deficient mice exhibited muscle atrophy, we examined gene expression of *MuRF1* and *Atrogin1*, which are markers of muscle atrophy. In the gastrocnemius muscle, but not in the tibialis anterior or soleus muscles, there was a significant increase in *MuRF1* and *Atrogin1* gene expression in mKO mice compared to Con mice (Supplementary Figure S3). In addition, the grip strength of the mKO mice was significantly lower than that of the Con mice (Figure 2E). These results suggest that post-developmental proteasome dysfunction induces muscle atrophy.

### Muscle-Specific Rpt3-Deficient Mice Exhibit a Decrease in the Muscle Fiber Size

**FIGURE 3:**
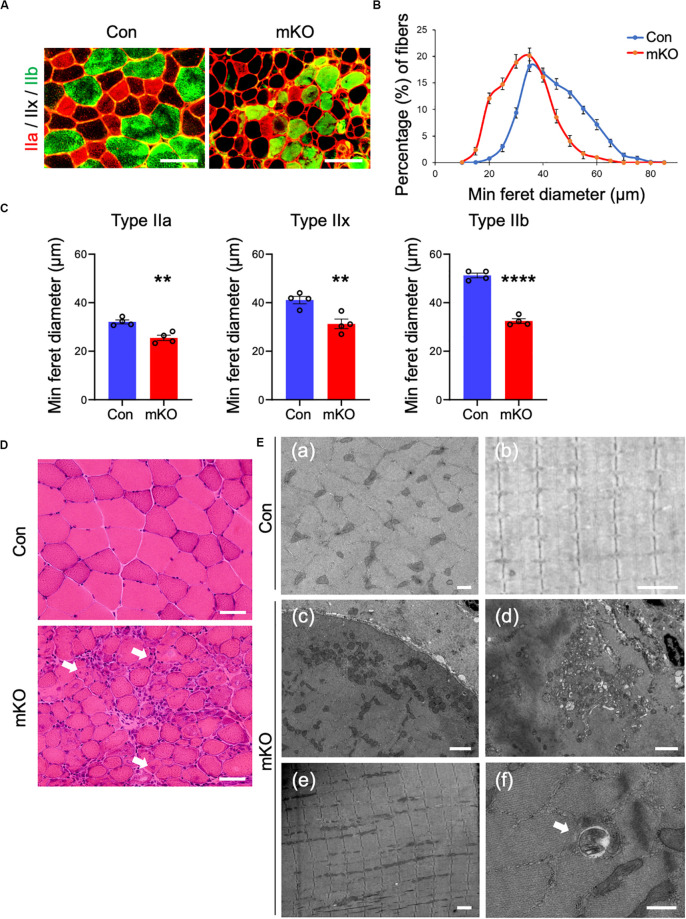
Muscle fiber sizes in muscle-specific Rpt3-deficient mice. **(A)** Immunohistochemical staining for myosin heavy chain type IIa (red), type IIb (green), and type IIx (unstained) in the tibialis anterior muscles of Con and mKO mice after doxycycline (DOX) treatment. Scale bar = 100 μm. **(B)** Distribution of muscle fiber minimal Feret’s diameters in the tibialis anterior muscles of Con and mKO mice. Data represent the means ± SEM (*n* = 4 per group). **(C)** Quantification of the minimal Feret’s diameter by the fiber type (IIa, IIx, and IIb) in the tibialis anterior muscles of Con and mKO mice. Data represent the means ± SEM (*t*-test: **p < 0.01, ****p < 0.0001; *n* = 4 per group). **(D)** H&E staining of the tibialis anterior muscles from Con and mKO mice. Arrows indicate the central nuclei in myofibers. Scale bar = 50 μm. **(E)** Electron micrographs of the tibialis anterior muscles from Con and mKO mice. (a) Cross-section of a muscle fiber. Scale bar = 500 nm. (b) Longitudinal section of a muscle fiber. Scale bar = 2 μm. (c) Cross-section of a muscle fiber. Normal mitochondria are located in the subsarcolemmal lesion. Scale bar = 2 μm. (d) Mitochondria and vacuolar structures in a necrotising fiber. Scale bar = 2 μm. (e) Longitudinal section of a muscle fiber. Scale bar = 2 μm. (f) Vacuolar structure in a non-necrotising fiber. Arrows indicate a vacuolar structure. Scale bar = 500 nm. Con indicates *Rpt3*^*f/f*^ mice, and mKO indicates muscle-specific *Rpt3*-knockout mice (*ACTA1-rtTA;tetO-Cre;Rpt3*^*f/f*^).

The fast-twitch fibers of the skeletal muscle are characterized by three types (type IIa, IIx, and IIb); type IIa fibers are oxidative, whereas type IIx and IIb fibers are mainly glycolytic. It has been reported that muscle atrophy varies with muscle fiber type (Wang and Pessin, 2013). Because the *Rpt3*-KO mice exhibited muscle atrophy, we next investigated the sizes of muscle fibers and fiber type in the Con and mKO mice. Immunofluorescence staining of cross-sections of the tibialis anterior muscles for myosin heavy chain type IIa/type IIb indicated that muscle fibers appeared smaller in the *Rpt3*-KO mice than in the Con mice (Figure 3A). Using a semi-automated measurement of the minimum Feret’s diameter of muscle cross-sections, we found that the myofiber sizes were smaller in the mKO mice than in the Con mice (Figure 3B). Moreover, we evaluated whether the extent of atrophy differed for different myofiber types and found that the sizes of type IIa, IIx, and IIb muscle fibers were significantly smaller in the mKO mice than in the Con mice (Figure 3C). Morphological observations revealed a general decrease in the sizes, along with the presence of central nuclei, in mKO myofibers (Figure 3D). Electron microscopy showed mitochondria and vacuolar structures in necrotising fibers in mKO mice (Figure 3E). Taken together, our data indicate that *Rpt3*-deficient mice exhibit decreased muscle fiber sizes.

### Effects of Proteasome Dysfunction on the Proteolytic System

**FIGURE 4:**
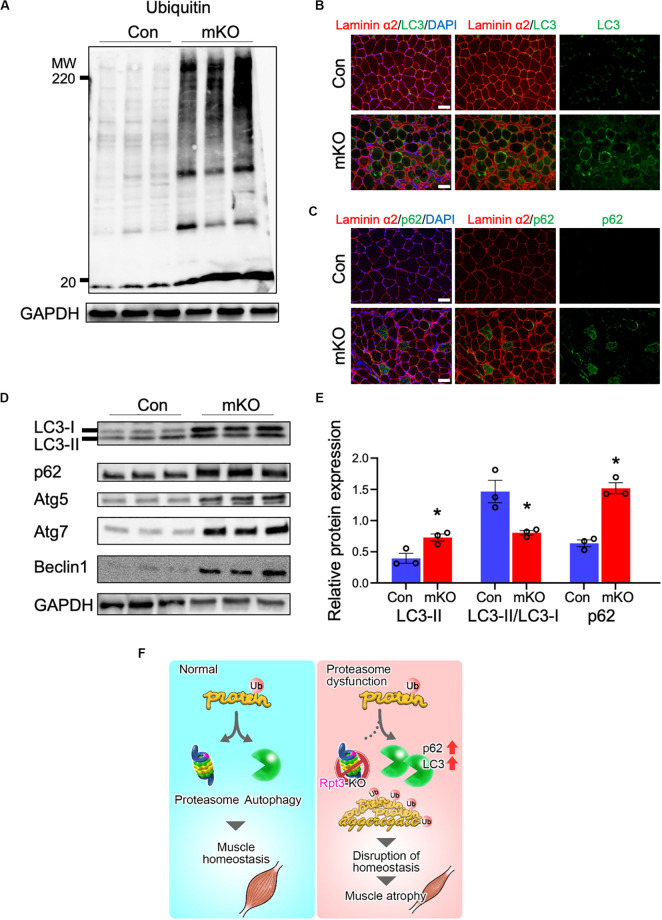
Effects of proteasome dysfunction on the proteolytic system. **(A)** Immunoblotting analysis of ubiquitinated proteins in the tibialis anterior muscles of Con and mKO mice after 3 weeks of doxycycline (DOX) treatment. GAPDH served as a loading control. **(B)** Immunostaining of cryosections of the tibialis anterior muscles of Con and mKO mice for laminin α2 (red), LC3 (green), and DAPI (blue) after 3 weeks of DOX treatment. Scale bar = 50 μm. **(C)** Immunostaining of cryosections of the tibialis anterior muscles of Con and mKO mice for laminin α2 (red), p62 (green), and DAPI (blue) after 3 weeks of DOX treatment. Scale bar = 50 μm. **(D)** Immunoblotting analysis of autophagy-related proteins in the tibialis anterior muscles of Con and mKO mice after 3 weeks of DOX treatment. **(E)** Quantification of LC3II and p62 proteins and the LC3II/LC3I ratio. Data represent the means ± SEM (*t*-test: *p < 0.05; *n* = 3 per group). **(F)** Graphical summary of the study. Con indicates *Rpt3*^*f/f*^ mice, and mKO indicates muscle-specific *Rpt3*-knockout mice (*ACTA1-rtTA;tetO-Cre;Rpt3*^*f/f*^).

The UPS degrades most of long- and short-lived normal and abnormal intracellular proteins (Goldberg, 2003; Collins and Goldberg, 2017). Because most proteasomal substrates must be ubiquitinated before degradation (Schrader et al., 2009), we next analyzed ubiquitinated proteins in the tibialis anterior muscles by immunoblotting and, as expected, found that their amounts were markedly higher in *Rpt3*-KO mice than in the Con mice (Figure 4A).

The autophagy pathway is also an important mechanism governing the degradation of proteins (Klionsky et al., 2016). LC3, which is a post-translational modifier, is required for autophagosome formation (Mizushima and Yoshimori, 2007). The p62 protein is involved in the aggregation of intracellular ubiquitin-associated proteins (Komatsu et al., 2007). Immunohistochemical analysis revealed higher levels of the LC3 and p62 proteins in the tibialis anterior muscles of the mKO mice than in those of the Con mice (Figures 4B,C). Next, autophagy-related proteins were evaluated by immunoblotting, and the data showed that the LC3II, p62, Atg5, Atg7, and Beclin-1 levels were higher and the LC3II/LC3I ratio showing autophagy flux was lower in the mKO mice than in the Con mice (Figures 4D,E). Taken together, these data suggest that proteasome-deficient mice had increased autophagosome formation but could not handle the accumulated proteins to be degraded.

## Discussion

In this study, we demonstrated that proteasome dysfunction in adult skeletal muscles induced muscle atrophy in a mouse model. Ablation of *Rpt3* in the skeletal muscle impaired proteasome-mediated proteolysis and led to muscle wasting and a decrease in the size of myofibers. In our previous study using *Mlc1f-Cre*;*Rpt3*^*f/f*^ mice, we found that proteasome function is required for muscle growth (Kitajima et al., 2014). To better understand the role of the proteolytic system in adult skeletal muscles, a mouse model with post-developmental proteasome dysfunction was established.

Morphological observations in proteasome-deficient muscles showed myofiber degeneration, including the presence of central and abnormal myonuclei. The UPS degrades most of the normal and abnormal intracellular proteins (Goldberg, 2003; Collins and Goldberg, 2017). Especially in muscle, proteolysis by the UPS is a major mechanism involved in myofibrillar protein degradation (Attaix et al., 1998; Hasselgren et al., 2002). Furthermore, while examining proteasome activity during regeneration, we found that it increased (Kitajima et al., 2018), indicating that maintenance of muscle mass, involving muscle regeneration/degeneration, is associated with the activation of the machinery involved in protein degradation. In muscle, an association between proteasome abnormalities and muscle disease has also been reported (Fratta et al., 2005; Askanas and Engel, 2006). Therefore, further research related to muscle proteasome dysfunction and disease is needed, for which the mouse model developed in the present study could be useful. Interestingly, in mKO mice, proteasome-related genes other than *Rpt3* were upregulated, similar to our previous observations in *Mlc1f-Cre*;*Rpt3*^*f/f*^ mice (Kitajima et al., 2014). These findings reflect the adaptations in Rpt3-deficient muscle to eliminate misfolded proteins more efficiently.

There have been several reports on the relationship between proteasome function and longevity. Specifically, it has been reported that proteasome activity is reduced with aging in the brain (Zeng et al., 2005), liver (Hayashi and Goto, 1998; Dasuri et al., 2009), heart (Bulteau et al., 2002), and muscle (Ferrington et al., 2005). Overexpression of proteasome subunits in yeast and *Caenorhabditis elegans* results in increased proteasome activity and prolonged lifespan (Chen et al., 2006; Vilchez et al., 2012). On the contrary, decreased proteasome activity in flies and mice has been reported to be associated with a shorter lifespan (Tonoki et al., 2009; Tomaru et al., 2012). Investigating the influence of muscle proteasome dysfunction on longevity would be an interesting topic for further studies. Moreover, a previous study reported the enhancement of proteasome activity through inhibition of USP14, a proteasome-associated deubiquitinating enzyme (Lee et al., 2010). It will be interesting to determine whether the proteasome activators also reduce muscle atrophy in mKO mice.

In this study, the tibialis anterior and gastrocnemius muscles, which consist of fast-twitch fibers, were markedly atrophied in mice with proteasomal dysfunction, whereas the slow-twitch-fiber soleus muscle did not show atrophy. This may be due to differences in the knockout efficiency of *Rpt3* gene expression. In fact, *Rpt3* gene expression in the tibialis anterior and gastrocnemius muscles was suppressed by more than 90%, whereas it was suppressed by less than 50% in the soleus muscle. Furthermore, the levels of proteasome-associated genes were significantly increased in the tibialis anterior and gastrocnemius muscles, but not in the soleus muscle. Knockout of *Rpt3* also caused a significant increase in proteasome activity in the tibialis anterior and gastrocnemius muscles. These results suggest that absence of *Rpt3* in the tibialis anterior and gastrocnemius muscles induces a feedback increase in the expression levels of proteasome subunits. In the soleus muscle, the phenotypic difference from the tibialis anterior and gastrocnemius muscle may have been due to inadequate suppression of *Rpt3* gene expression. Taken together, *Rpt3* deficiency disturbed proper degradation of ubiquitinated proteins in proteasome and that seemed to result in compensatory upregulation of other proteasome-related genes. Because proteasome function declines with age (Bardag-Gorce et al., 1999; Ferrington et al., 2005), proteasome insufficiency may be partially related to sarcopenia which is an age-related loss of muscle mass and strength (Musaro and Scicchitano, 2019). Our mouse model may help to further understanding of the underlying mechanism of the decline in physical performance in the elderly.

Two muscle-specific E3 ubiquitin ligases, MuRF1 and Atrogin1, are thought to be key regulators of proteasomal proteolysis in skeletal muscle, especially under atrophy-inducing conditions (Cai et al., 2004; Sandri et al., 2004). In this study, when examining their gene expression in the gastrocnemius and tibialis anterior muscles that exhibited muscle atrophy, we found a significant increase in gene expression only in the gastrocnemius muscle. This difference in gene expression between the gastrocnemius and tibialis anterior muscles may be due to the timing of sampling. In fact, as shown in Figures 1D,E,G,H, proteasome-related genes were increased more in the gastrocnemius muscle compared to the tibialis anterior muscle. A study examining *MuRF1* and *Atrogin1* gene expression in several models of muscle atrophy has also reported that their gene expression is up-regulated at different time points (Bodine et al., 2001). In this study, an increase in *MuRF1* and *Atrogin1* gene expressions in the tibialis anterior muscle could be confirmed by examining the gene expression in several time courses.

Previous studies have shown that inhibition of the proteasome system induces autophagy *in vivo* and *in vitro* (Iwata et al., 2005; Pandey et al., 2007; Zhu et al., 2010). In this study, the *Rpt3*-deficient mice exhibited an increase in the expression levels of the p62 and LC3 proteins, suggesting the activation of autophagy. Immunostaining also detected apparent accumulation of p62 and LC3 in myofibers and elevation of the LC3II protein level in muscles of the mKO mice. As LC3II is a lipidated form of LC3 (Kabeya et al., 2000), these results indicated an increase in autophagosome formation. In a previous study, inhibition of the proteasome by specific silencing of a proteasome subunit also stimulated increases in Atg5 and Atg7 levels (Zhu et al., 2010). Consistently, the levels of Atg5, Atg7, and Beclin-1, which are involved in the formation of the isolation membrane, were elevated in the mKO muscle in this study. Thus, in adult muscle-specific proteasome-deficient mice, proteasome suppression caused an increase in autophagic protein degradation; however, this increase could not fully compensate for the loss of *Rpt3* to maintain proteolytic system, resulting in skeletal muscle atrophy (Figure 4F).

Our results showed that the proteasome suppression in adult muscle severely deteriorated myofiber integrity and led to muscle atrophy. Although a further study is needed to understand the role of adult skeletal muscles in various physiological processes that involve the proteasome system, this work lays the foundation for understanding the regulation of proteolysis in adult skeletal muscles.

## Data Availability Statement

The raw data supporting the conclusions of this article will be made available by the authors, without undue reservation.

## Ethics Statement

The animal study was reviewed and approved by The Experimental Animal Care and Use Committee of the Kumamoto University approved the animal experimentation (Ref. No. A30-098).

## Author Contributions

YK designed the experiments, performed the experiments, interpreted the data, assembled the input data, and wrote the manuscript. NS, KY, RI, and MT performed the experiments. YT and RT produced the animals. MA and YO interpreted the data and assembled the input data. All authors discussed the results and implications and commented on the manuscript.

## Conflict of Interest

The authors declare that the research was conducted in the absence of any commercial or financial relationships that could be construed as a potential conflict of interest.

## Supplementary Material

FIGURE S1 Expression of proteasome-related genes in the soleus muscles of Con and mKO mice after DOX treatment. **(A)** Relative expression of *Rpt3* mRNA in the soleus muscles of Con and mKO mice after DOX treatment. The *Tbp* gene was used as an internal control. Data represent the means ± SEM (*n* = 4 per group). **(B)** Relative mRNA expression of 19S proteasome genes (*Psmd3, 4, 11, 13*, and *Rpt6*) in the soleus muscles of Con and mKO mice after DOX treatment. The *Tbp* gene was used as an internal control. Data represent the means ± SEM (*t*-test: ^∗∗^*p* < 0.01; *n* = 4 per group). **(C)** Relative mRNA expression of 20S proteasome genes (*Psmb2, 4, 5*, and *7*) in the soleus muscles of Con and mKO mice after DOX treatment. The *Tbp* gene was used as an internal control. Data represent the means ± SEM (*t*-test: ^∗^*p* < 0.05; *n* = 4 per group). Con indicates *Rpt3*^*f/f*^ mice, and mKO indicates muscle-specific *Rpt3*-knockout mice (*ACTA1-rtTA;tetO-Cre;Rpt3*^*f/f*^). AU, arbitrary units.

FIGURE S2 The chymotrypsin-like proteasome activities (relative to those in Con) in the soleus, tibialis anterior, and gastrocnemius muscles after DOX treatment are shown. Data represent means ± SEM (*t-*test: ^∗^*p* < 0.05, versus Con; *n* = 3–4 per group). IU, international units.

FIGURE S3 Relative expression of *MuRF1* and *Atrogin1* mRNA in the soleus, tibialis anterior, and gastrocnemius muscles of Con and mKO mice after DOX treatment. The *Tbp* gene was used as an internal control. Data represent the means ± SEM (*t*-test: ^∗∗∗^*p* < 0.001; *n* = 3–4 per group).

TABLE S1 Primers for quantitative RT PCR.
