# Supplementary figures and images for: Inducible Rpt3, a Proteasome Component, Knockout in Adult Skeletal Muscle Results in Muscle Atrophy
Source: Front Cell Dev Biol. 2020 Sep 2;8:859. doi: 10.3389/fcell.2020.00859 (PMC7492297; doi:10.3389/fcell.2020.00859)

# Supplementary Figure 1

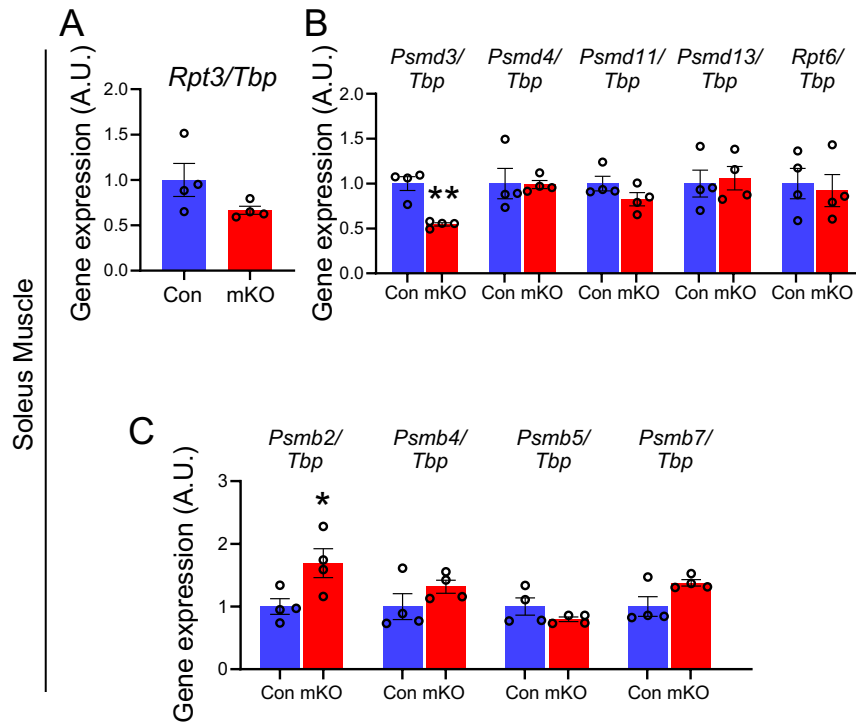

Supplement: FIGURE S1 — Expression of proteasome-related genes in the soleus muscles of Con and mKO mice after DOX treatment. (A) Relative expression of Rpt3 mRNA in the soleus muscles of Con and mKO mice after DOX treatment. The Tbp gene was used as an internal control. Data represent the means ± SEM (n = 4 per group). (B) Relative mRNA expression of 19S proteasome genes (Psmd3, 4, 11, 13, and Rpt6) in the soleus muscles of Con and mKO mice after DOX treatment. The Tbp gene was used as an internal control. Data represent the means ± SEM (t-test: ∗∗p < 0.01; n = 4 per group). (C) Relative mRNA expression of 20S proteasome genes (Psmb2, 4, 5, and 7) in the soleus muscles of Con and mKO mice after DOX treatment. The Tbp gene was used as an internal control. Data represent the means ± SEM (t-test: ∗p < 0.05; n = 4 per group). Con indicates Rpt3f/f mice, and mKO indicates muscle-specific Rpt3-knockout mice (ACTA1-rtTA;tetO-Cre;Rpt3f/f). AU, arbitrary units. [file Image_1.pdf]

## Supplementary Figure 2

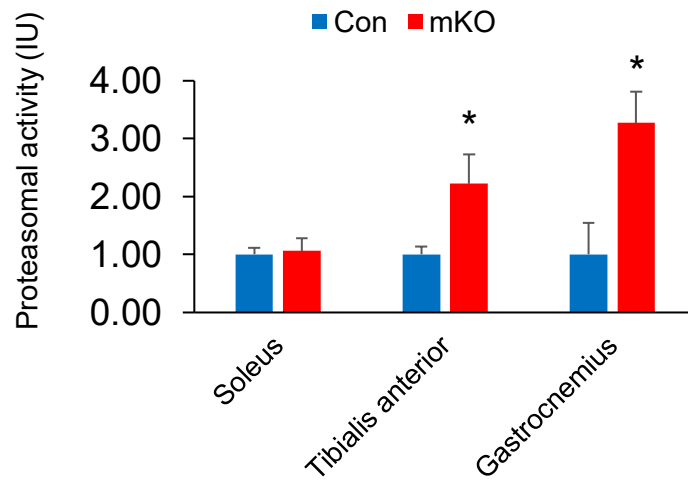

Supplement: FIGURE S2 — The chymotrypsin-like proteasome activities (relative to those in Con) in the soleus, tibialis anterior, and gastrocnemius muscles after DOX treatment are shown. Data represent means ± SEM (t-test: ∗p < 0.05, versus Con; n = 3–4 per group). IU, international units. [file Image_2.pdf]

# Supplementary Figure 3

*Rpt3<sup>fl/fl</sup>* (Con)  
*ACTA1-rtTA;tetO-Cre; Rpt3<sup>fl/fl</sup>* (mKO)

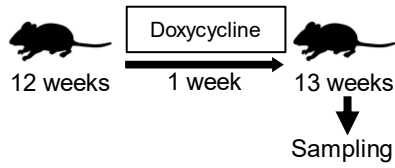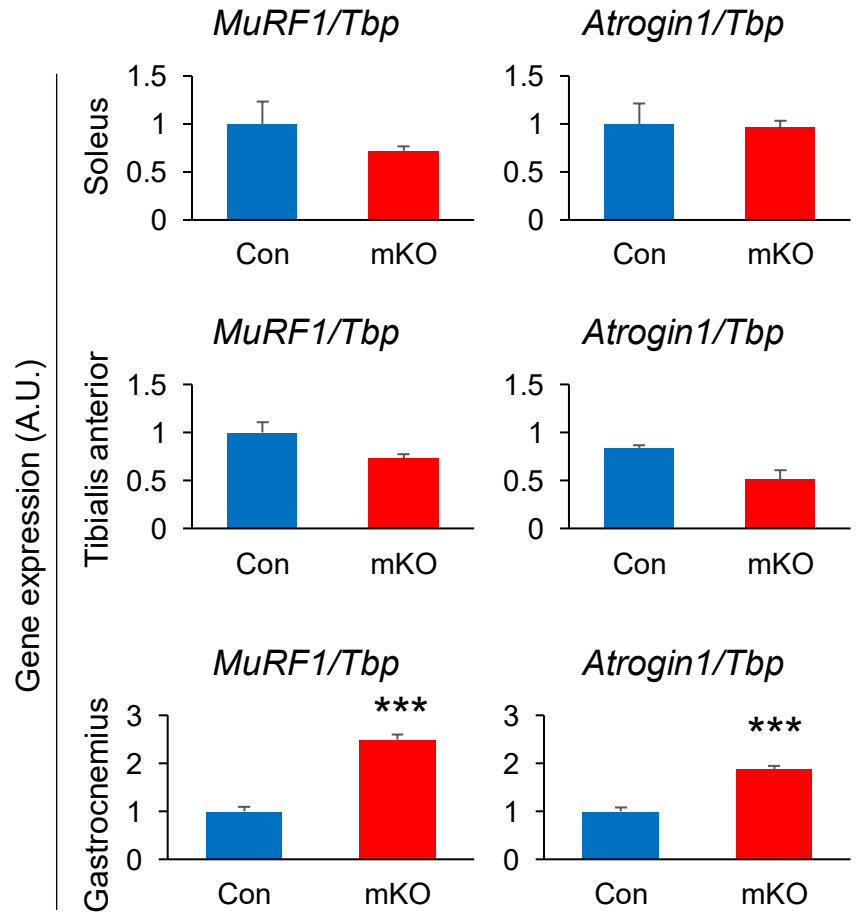

Supplement: FIGURE S3 — Relative expression of MuRF1 and Atrogin1 mRNA in the soleus, tibialis anterior, and gastrocnemius muscles of Con and mKO mice after DOX treatment. The Tbp gene was used as an internal control. Data represent the means ± SEM (t-test: ∗∗∗p < 0.001; n = 3–4 per group). [file Image_3.pdf]
