# Supplementary material for: Inducible Rpt3, a Proteasome Component, Knockout in Adult Skeletal Muscle Results in Muscle Atrophy
Source: Front Cell Dev Biol. 2020 Sep 2;8:859. doi: 10.3389/fcell.2020.00859 (PMC7492297; doi:10.3389/fcell.2020.00859)
Supplement: TABLE S1 — Primers for quantitative RT PCR. [file Table_1.pdf]

Table 1. Primers for quantitative RT PCR

Rpt3-F	CTACAGGCCTTGCTCAGCTC	Rpt3-R	AGATTGTGGGCGTCTGTAGG
Tbp-F	CAGATGTGCGTCAGGCGTTC	Tbp-R	TAGTGATGCTGGGCACTGCG
Psmd3-F	ACCAGGCTGAGAAGCTGGTA	Psmd3-R	AACAGCTGTGTGCTGTGGAG
Psmd4-F	CTTGGTGCCAGTGACTTTGA	Psmd4-R	GTAGCAATTCCAGCCTCAGC
Psmd11-F	GAATGGGCCAAATCAGAGAA	Psmd11-R	CCACCAAAAGGGCTTTATCA
Psmd13-F	TCCAGCTGTTGTGCCTTATG	Psmd13-R	TCCTTCATCCCCTTGATCTG
Rpt6-F	GTGGACCCTTTGGTGTCACT	Rpt6-R	GCCAACAATGTCTTCCCAGT
Psmb2-F	CTGGCTATGACGAGCATGAA	Psmb2-R	TGGAGCTCCTCCAGACACTT
Psmb4-F	TTATGCGAGTCAACGACAGC	Psmb4-R	CTTTCTCCGTCAGCATAGCC
Psmb5-F	ATCGAAATGCTTCACGGAAC	Psmb5-R	CGTTCCTTATTGCGAAGCTC
Psmb7-F	GTGTCGGTGTTTCAGCCAC	Psmb7-R	TCCGCTTCCAAGACAGCATTC
MuRF1-F	ACCTGCTGGTGGAAAACATC	MuRF1-R	CTTCGTGTTCCTTGCACATC
Atrogin1-F	ATGCACACTGGTGCAGAGAG	Atrogin1-R	TGTAAGCACACAGGCAGGTC
